# Combating the global spread of poverty-related Monkeypox outbreaks and beyond

**DOI:** 10.1186/s40249-022-01004-9

**Published:** 2022-07-07

**Authors:** Ernest Tambo, Ashwaq M. Al-Nazawi

**Affiliations:** 1 Africa Disease Intelligence, Preparedness and Response, Yaoundé, Cameroon; 2 Depatment Public Health, Faculty of Medicine, Universite des Montagnes, Bangangte, Cameroon; 3 Vector-Borne and Zoonotic Diseases Department, Public Health Directorate, Ministry of Health, Jeddah, 22246 Saudi Arabia

**Keywords:** Monkeypox, Outbreak, Neglected tropical diseases, Poverty, One Health, Preparedness, Emergency response, Vaccination, Research

## Abstract

The current unprecedented Monkeypox outbreaks emergence and spread on non-endemic countries has led to over 3413 laboratory confirmed cases and one death, and yet, does not constitute a public health emergency of international concern as June 23th 2022. We urgently call for collective regional and global partnership, leadership commitment and investment to rapidly strengthen and implement Monkeypox World Health Organization outbreak Preparedness and emergency response actions plans implementation against Monkeypox outbreak. Given the importance of human–animal–environment interface and transmission dynamics, fostering global and regional One Health approach partnership and multisectoral collaboration programs have timely and robust sustained investment benefits on poverty-linked Monkeypox and other emerging epidemics population-based programs, while leveraging from lessons learnt. Moving forward requires addressing priority research questions listed and closing the knowledge gaps for Monkeypox and others neglected tropical diseases roadmap implementation in vulnerable and at risk countries.

## Background

Since the first human Monkeypox case reported in 1970 in Demographic Republic of Congo, poverty related Monkeypox cases have been concentrated in tropical rain forest regions of Central and West Africa. The emerging Monkeypox outbreak has been reported in 35 non-endemic World Health Organization (WHO) members’ countries with more than 3413 laboratory testing confirmed cases and one death as at June 27th, 2022. The unprecedented geographical Monkeypox emergence and distribution and transmission dynamic showed the majority of cases (*n* = 2933, 86%) in WHO European regions and the American region (*n* = 381, 11%), Eastern Mediterranean (*n* = 13, < 1%) and Western Pacific (*n* = 7, < 1%) [1]. The occurrence of multiple outbreak globally could constitute a ‘pandemic potential’, but there is still a window of opportunity to prevent onward widespread. As far, WHO expert committee resolved by consensus to advise the WHO Director-General that at this stage the current outbreak of Monkeypox should not constitute a public health emergency of international concern on June 23th [1]. However, the emergency nature of the event and that controlling the further spread of outbreak requires collective and coordinated evidence-based response and actions efforts [1]. These include enhanced preparedness and surveillance, and contact-tracking, close monitoring and prompt care of patients, effective information sharing and risk communication, access to and use of most effective tools based on experts’ guidance and technical assistance under the International Health regulations (2005).

So far, six endemic African countries linked to poverty associated factors and animal reservoirs have reported 1365 cases (including suspected cases) and 1 death due to the same virus from mid-December to June 15th 2022 1]. It should be noted that Monkeypox is known to be endemic and appear circulating below levels detectable by routine surveillance and sustained direct and/or indirect human–human transmission in 11 African countries: Benin, Cameroon, the Central African Republic, the Democratic Republic of the Congo, Gabon, Cote d’Ivoire, Liberia, Nigeria, the Republic of the Congo, Sierra Leone and South Sudan [1, 2], but less severe with a case fatality rate ranging from 3.6% and 10.6% in 2017 [2]. Yet, the number of cases grossly underestimated due to lack or poor laboratory surveillance/weak routine detection and others confounders including asymptomatic infection, sexual transmission and the role of mass gatherings in sub-Saharan Africa and elsewhere.

On 19th May, Portugal reported the first draft genome sequence of the Monkeypox virus associated with this major outbreak affecting multiple countries worldwide. Until 8th June, cases of 16 countries indicated the 2022 virus belongs to the West African clade and is most closely related to viruses associated with the exportation of Monkeypox virus from Nigeria to several countries in 2018 and 2019, namely the United Kingdom, Israel and Singapore [1, 2]. But, it segregates in a divergent phylogenetic branch, likely reflecting continuous accelerated evolution [4]. Indeed, additional apolipoprotein B mRNA editing enzyme, catalytic subunit G (APOBEC3) type mutations have been observed within 8 genomes sampled from the outbreak in Portugal including 2 that are shared by 2 genomes suggesting ongoing transmission of these. These suggest further research including sequencing and analysis of within-host variation in APOBEC3 editing to investigate its potential for controlling or moderating Monkeypox viral human infections [5]. It is also a crucial issue to think about what level of concerns against Monkeypox needs to be considered now and beyond local and global concern?

## Current status of global and regional efforts against Monkeypox outbreaks

The emergence and re-emergence of new and old infectious diseases has widely been linked to poverty and vulnerability, malnutrition and poor healthcare behavior that is more prevalent in low-income countries mainly in sub Saharan Africa. Previous studies and reports have showed a significant relationship between poverty and vulnerability to poverty related outbreak or infectious outbreak emergence and spread in developing and developed countries [3]. This emergence of Monkeypox in over 50 high income countries has been probably associated with multiple and unprotected sexual activities amongst young age/adults, contact with infected patient, infected tools and products or untreated HIV/compromised immunodeficiency infections respectively. The disease can be transmitted directly or indirectly through contact with infected skin lesions of contaminated patients, share towels and bedding, gays and unprotected bisexual intercourse. Typical symptoms varied from fever, aches, myalgia, intense headache, lymphadenopathy and skin eruption similar to Chickenpox, syphilis and herpes simplex [1, 3].

Although the factors influencing the current Monkeypox outbreak in non-endemic areas are unclear. However, the COVID-19 pandemic, unemployment, international trade and travel have an impact on the cross-country transmission of Monkeypox. Moreover, the lack of surveillance and detection capacity due to poverty in endemic areas affects the prevention and control response to Monkeypox. Yet, ongoing Monkeypox outbreak emergence and rapid spread in non-endemic countries is still poorly understood. However, global travel and trade, job loss and COVID-19 pandemic economic and social conditions effects to unhealthy sexual and reproductive activities have been linked to the ongoing outbreak and transmission in non-endemic developed countries. It has long be recognized that poverty is one of the major social and economic determinants of health, hunger, ill-health and poor environment, inadequate sanitation and poor potable drinking water related vulnerability amongst at risk groups. Since the poor populations face a higher spark risk and spread related health and economic shocks, understanding why and how the poor or rich are more vulnerable to neglected tropical disease outbreak and pandemic burden is crucial. Analyzing and understanding aspects of what protections are required and how they affect individuals and societies due to under investment in health system preparedness, lack of effective Monkeypox vaccine and therapies, weaker health systems, limited capacity and how impoverishment reflects the painful choices facing poor African countries at greatest risk, undetected longer and smolder spread will go far in aiding address the increasing outbreaks dynamics around management and control of outbreaks [3, 4].

It has been demonstrated that poverty in time of pandemic or epidemic infection rates showed no significant difference under non-intervention, the peak case is maximized across poor and rich people. Also, larger populations, higher fraction of poor and long durations of interventions are found to progressively worsen outcomes for the poor mainly stigmatization and social deprivations. Addressing Monkeypox outbreak poverty related vicious cycle and challenges requires a deep and rigorous operational research to understanding structural poverty and epidemic. Syndemic or comorbidity of COVID-19 and ubiquitous malnutrition linked to rapid expansions of human settlements and climate change, intensifying livestock production and amplifying outbreaks threats continue to bear the brunt and a toll on vulnerable and marginalized groups in Africa and others low income countries. Noting the absence of regular and sustained Monkeypox surveillance and monitoring mechanisms in both endemic and non-endemic countries, the syndemic of COVID-19 pandemic and Monkeypox epidemic could be of more significant health, socioeconomic and global health security consequences if effective and sustained programs and interventions failed to respond to poverty vicious cycle needs amongst the most vulnerable and marginalized groups.

Ongoing epidemiological investigation reported that most cases are linked to men–men sexual intercourse and have no travel history in endemic settings. Hence, non-travel reported case is atypical and could serve as outbreak alertness and strengthening of international health regulations 2005 and global health security measures [1]. The unexpected Monkeypox outbreak spread in new zones remain of global and regional concern to forthcoming two major mass gatherings events will be taking place in Gulf countries namely the Hajj and Umrah 2022 in Saudi Arabia and Qatar FIFA World Cup 2022 slated to over 2 million participants, tourists and visitors. Hence, proactive and precautionary measures including traveler information update and appropriate vaccinations implementation are vital responses and countermeasures to prevent and contain the threats and post-event effects.

## Preparedness and emergency response against Monkeypox through more collaboration

Hence, we urgently call for collective partnership and action by global and regional leadership and investment commitment to rapidly strengthen and implement Monkeypox WHO outbreak preparedness and response actions plans, neglected tropical diseases (NTDs) roadmap operationalization for contextual and practical evidence-based guidelines and actions [3, 5]. These is critical in tackling and containing the emerging and persisting poverty-linked global Monkeypox outbreak and ongoing COVID-19 pandemic response and recovery efforts. These rely on innovative and contextual public health preparedness and emergency response, and recovery strategies that encompass: (i) scaling up regional and global partnership and coordination of epidemiological surveillance including point of entries outbreak surveillance, case investigation and contact-tacking, and timely information sharing and risk communication for travel information guide update, monitoring of animal and human populations to spot outbreaks and contain it quickly, (ii) enhanced poverty alleviation strategies implementation and a range of investment packages, (iii) mount early and effective risk communication and community engagement programs, (iii) scale diagnostic testing and contact tracing, (iv) Enhanced rash-like illnesses genomic surveillance and comprehensive case finding, infection prevention and control measures at all levels mainly healthcare delivery services sites, (v) promoting local and international resilient health security workforce against Monkeypox and others infectious diseases threats, (vi) improving clinical management with smallpox antivirals and supportive care, (vii) boosting mass gatherings including pilgrims and tourist/traveler-related information update and strict quarantine measures regularly.

Thus, Monkeypox outbreak and COVID-19 pandemic risk communication and community engagement, building outbreak communities’ resilience and containment interventions should preferentially target at risk and marginalized populations in endemic countries, equal protection to all citizens whether poor or rich. Investing in equitable access to novel tools including vaccines and medicines, food security and nutrition, are critical factors influencing health and behavior in response and recovery programs. These efforts contribute in upholding national and regional health security and capacity to prevent, detect and respond to poverty related diseases outbreaks and pandemics in both rural and urban settings. Scaling up access and adherence to critical services to HIV/TB, Cancer and others immune-depressive diseases, to building capacity on early warning alerts, preparedness and emergency response pro-activeness and response to curb and stop the onward Monkeypox widespread.

## Role of One Health approach against zoonotic Monkeypox outbreak

Given the importance of human–animal–environment interface in the emergence and spread of outbreak, fostering One Health approach activities and multisectoral collaboration have benefits through timely and robust sustained investment on Monkeypox and other emerging epidemics population-based programs to reduce poverty, while leveraging from lessons learnt from others pandemics or outbreaks implementation. Increasing the most vulnerable poor health education and awareness outreach, coupled with evidence-based COVID-19 response and recovery programs, financial mechanisms and interventions, community health workers training and capacity building in COVID-19 and other routine vaccination programs efficient and sustainable delivery is needed. In addition, increasing effective mitigating and information communication against the social media rumors, and misinformation to prevent and control the growing Monkeypox outbreak coupled with COVID-19 pandemic public and global health effects [2, 6]. Furthermore, operational One Health research agenda on poverty related outbreaks and pandemic should be initiated in improving local and regional surveillance, preparedness and response interventions, in contributing to global health security agenda.

Strengthening International Health regulations and global security actions against Monkeypox outbreak, should include effficient preventative and precautionary measures, prompt clinical management and vaccines research collaboration. Most importantly, fostering One Health approach implementation, are crucial implementation after understanding Zoonotic viral diseases including Monkeypox and COVID-19 animal reservoirs–human link, environment transmission and interconnections for evidence based solutions. Interestingly, two smallpox vaccines (ACAM-2000 and MVA-BN) have been recommended for vaccination against Monkeypox to people at risk of occupational exposure (Laboratory technician, clinical workers, frontline staff) by the European Medicines Agency in 2013 and US Food and Drug Administration (FDA) in 2019 respectively [2, 6]. However it efficacy and safety of these vaccines approved still require further clinical trials and pharmacovigilance monitoring amongst vulnerable groups to guarantee herd immunity against this viral infection. These include better diagnostic for surveillance, improved hospital infection prevention and control measures, and social and safety containment countermeasures. These are required joint actions implementation to curtail the outbreak by securing equitable risk and information sharing and public engagement, widely access to vaccines, vaccine distribution, and effective contextual risk communication and engagement strategies to target Monkeypox vaccination coverage amidst COVID-19 pandemic response to the most vulnerable and marginalized poor and rich people.

Prompt, reliable and secured information sources ought to continue rise citizens and country awareness and education outreach, healthcare decentralization and transparency for readiness by governments and stakeholders to prevent, monitor and tackle any eventual outbreak of Monkeypox. Country-led poverty alleviation strategies and efforts in improving effective partnership and governance, strengthening quality care delivery services, vaccination stockpile, coverage and effectiveness are vital for resilience building and improved health outcomes. These are required to prepare and strengthen at risk and marginalized communities, enhanced empowerment and knowledge of all risk populations including travelers, pilgrims and tourists, to such unprecedented global outbreak effects on travel medicine, global trade and health agenda [1, 7, 8]. More operational research is needed in building evidence for comprehensive and efficient decisions policies and public engagement targeted actions. It is crucial to address knowledge gaps and poverty linked outbreak and NTDs roadmap and research priorities 2030 [1, 2]. This is paramount to improve available data and information to build evidence-based decision making policies, effective and sustainable countermeasures, and innovative response solutions to prevent and contain these emerging and reemerging infectious diseases outbreaks in old and new zones.

## Moving forward and research priorities

To address what level of concerns need to be taken for controlling Monkeypox outbreak, in the wake of the current outbreak in non-endemic and endemic regions, It is essential to close the knowledge gaps and address priority research questions for Monkeypox and other NTDs operational research and roadmap implementation [9] including:

i. Contagiousness and transmission routes, Is the incubation period really non-infectious? Interpersonal transmission routes and patterns (aerosol transmission, droplet transmission, mother-to-child transmission, sexual transmission)? Detoxification pattern and its impact on transmission? The length of the infectious period and its influencing factors, and the possibility of cross-species transmission?
ii. Natural history of the disease: clinical manifestations, severity, organs involved and pathological effects, influencing factors.
iii. Co-infections: Infection status with chickenpox, sexually transmitted diseases, malaria, etc.
  Better understanding of the impact on virus transmission and disease severity (pathophysiology) could focus on:
iv. Clinical treatment: Optimal regimen, long-term effects and prognosis (mid- to long-term comorbidities, sequelae and stigma, special populations).
v. Disease prevention and control: Virus viability of external environment and object surfaces, disinfection methods, optimal ventilation protocols, optimal isolation protocols, effluent monitoring.
vi. Occupational population prevention and control: infection status of occupational population, risk assessment of occupational exposure and corresponding means of prevention, evaluation of the effectiveness of vaccines and drugs used after exposure, vaccination of occupational population and effective waste management.

## Abbreviation

NTDs: Neglected tropical diseases
